# Farrerol prevents Angiotensin II-induced cardiac remodeling *in vivo* and *in vitro*

**DOI:** 10.3389/fphar.2022.1079251

**Published:** 2023-01-04

**Authors:** Jian He, Dengyue Xu, Lu Wang, Xiaohong Yu

**Affiliations:** ^1^ Department of Cardiovascular Surgery, The First Affiliated Hospital of Dalian Medical University, Dalian, China; ^2^ Department of Cardiovascular Surgery, General Hospital of Northern Theater Command of China Medical University, Shenyang, China; ^3^ Department of Cardiology, The First Affiliated Hospital of Dalian Medical University, Dalian, China

**Keywords:** Farrerol, myocardial remodeling, inflammation, fibrosis, oxidative stress

## Abstract

Cardiovascular disease has become the primary disease that threatens human health and is considered the leading cause of death. Cardiac remodeling, which is associated with cardiovascular disease, mainly manifests as cardiac hypertrophy, fibrosis, inflammation, and oxidative stress. Farrerol plays an important role in treating conditions such as inflammation, endothelial injury and tumors, and we speculated that Farrerol may also play an important role in mitigating cardiac hypertrophy and remodeling. We established a model of myocardial remodeling using Angiotensin II (Ang II) with concurrent intraperitoneal injection of Farrerol as an intervention. We used cardiac ultrasound, immunohistochemistry, Immunofluorescence, Wheat Germ Agglutinin, Dihydroethidium, Western Blot, qPCR and other methods to detect the role of Farrerol in cardiac remodeling. The results showed that Farrerol inhibited Ang II-induced cardiac hypertrophy; decreased the ratio of heart weight to tibia length in mice; reduced inflammation, fibrosis, and oxidative stress; and reduced the size of cardiomyocytes *in vivo*. Farrerol inhibited Ang II-induced cardiomyocyte hypertrophy, levels of oxidative stress, and the proliferation and migration of fibroblast *in vitro*. Our results revealed that Farrerol could inhibit Ang II-induced cardiac remodeling. Farrerol may therefore be a candidate drug for the treatment of myocardial remodeling.

## 1 Introduction

Cardiovascular disease has become the primary disease that threatens human health and is considered a leading cause of death (Zhang et al., 2019). Heart failure is the final outcome in a variety of cardiovascular diseases, such as hypertension, coronary heart disease, and cardiomyopathy. Although existing clinical drugs, device therapies and adjuvant treatments can delay the progression of heart failure, the overall prognosis of patients with heart failure is still very poor. Myocardial remodeling is a major component of heart injury and is a pathophysiological process that occurs when cardiovascular disease progresses to heart failure (Aimo et al., 2019; Maki and Takeda, 2020).

Cardiac remodeling refers to adaptive and compensatory changes in the structure, morphology and function of the heart in response to various pathophysiological factors (Montiel et al., 2020; Nadruz, 2015; Porter and Turner, 2009). It is a serious pathological change in the development of heart failure. Cardiac remodeling mainly entails structural remodeling and electrical remodeling. The main features are myocardial interstitial fibrosis, cardiomyocyte hypertrophy, reduced ventricular compliance and abnormal ventricular electrical conduction, which together can directly affect cardiac function and even lead to death (Dixon and Spinale, 2011). Therefore, the prevention and treatment of heart failure through the mitigation of myocardial remodeling holds great importance and potential.

Epidemiology and molecular biology studies have shown that flavonoids can reduce the risk of cardiovascular disease in the diet, and that their biological functions may be related to antioxidant effects (Dludla et al., 2017). Farrerol is the main ingredient isolated from the traditional Chinese herbal medicine Manshanhong (Yin et al., 2019).

Recent studies have shown that Farrerol has various biological functions such as scavenging free radicals, regulating enzyme activity, inhibiting cell proliferation and inflammatory responses. Farrerol has some potential therapeutic effects in a variety of diseases including cardiovascular disease, neurological disease, metabolic disease and cancer (Chae et al., 2021; Duan et al., 2021; Li et al., 2019). Studies have shown that Farrerol can inhibit oxidative stress and inflammatory reactions, thereby reducing microglial cell damage (Cui et al., 2019). Farrerol can reduce the damage of renal mesangial cells induced by high sugar Chen et al. (2020). Farrerol can relieve collagenase-induced tendinopathy by inhibiting ferroptosis Wu et al. (2022). Farrerol effectively inhibit cisplatin-induced inflammation and renal fibrosis by activating Nrf2 and PINK1/Parkin-mediated mitophagy (Ma et al., 2021). Farrerol suppress the migration, invasion, and induce the apoptosis of LSCC cells *via* the mitochondria-mediated pathway (Zhang et al., 2021). Farrerol can reduce the oxygenation damage of the endothelium in the blood duct (Yan et al., 2020). However, there have been no studies published on cardiac remodeling.

One biologically active octapeptide involved in remodeling is Angiotensin II (Ang II), which is found in the renin-angiotensin aldosterone system (RAAS). Ang II functions mainly through angiotensin type 1 receptor (AT1 receptor, AT1R) and angiotensin type 2 receptor (AT2 receptor, AT2R). AT1R mediated most of the physiological and pathological effects of Ang II. The AT1R is the most well studied angiotensin receptor. When activated by Ang II, AT1R activates phospholipase C (PLC) and increases Ca^2+^ concentration, or inhibits adenylate cyclase, which activates a series of cellular reactions, like activating reactive oxygen species (ROS)-dependent molecular signaling pathways or TAK1-mediated cardiac inflammation (Dai et al., 2022; Ding et al., 2020; Rao et al., 2020). Activation of AT1 receptor can cause vasoconstriction, aldosterone synthesis and secretion, increase vasopressin secretion, promote myocardial hypertrophy, increase peripheral norepinephrine activity, proliferation of vascular smooth muscle cells, inhibit renin secretion, affect cardiac contractility and extracellular matrix synthesis (Lin et al., 2022; Bhullar and Dhalla, 2022; Booz et al., 2021; Mondaca-Ruff et al., 2022).

The aim of the experiment in the present study was to determine the effect of Farrerol on cardiac remodeling and cardiac hypertrophy induced by Ang II. The results showed that Farrerol could inhibit Ang II-induced cardiac hypertrophy and reduce HW/TL, inflammation, fibrosis, oxidative stress, the volume of cardiomyocytes and the proliferation and migration of fibroblast. The results from this experiment suggest that Farrerol may represent a new therapeutic target for cardiac remodeling.

## 2 Materials and methods

### 2.1 Animals

Male wild-type C57BL/6J mice, 8-weeks-old, purchased from Aikesaisi Biotechnology Co., Ltd., kept in an environmentally controlled room with a 12-h light/dark cycle. The animal experiment was approved by the Animal Research Committee of Dalian Medical University (AEE20029). Farrerol (Selleck, S9552, China) was dissolved in dimethyl sulfoxide (DMSO, Sigma), diluted with DMSO and normal saline, and injected intraperitoneally at a dose of 10 mg/kg/day (Ma et al., 2021; Zhou et al., 2022). 2 days after administration, Ang II (1,000 ng/kg/min, 4474-91-3, aladdin) was subcutaneously infused to establish a cardiac remodeling model for 2 weeks *via* implanted osmotic minipumps (Model 1007D, Alzet, Cupertino, CA, United States). This method is a recognized method to establish cardiac remodeling.

### 2.2 Echocardiography

The anesthetized mice lay on their backs on a platform with their paws placed on electrodes. Ensure appropriate ECG, body temperature was around 37°C. Vevo 2100 high-resolution imaging system (VisualSonics Inc.) was used to perform chest echocardiography on mice to measure ejection fraction (EF%), fractional shortening (FS%) and other indicators. Induce anesthesia with 3% isoflurane and maintain at 1%–2% during the procedure. Mice were immobilized and supine. A parasternal short-axis image of the left ventricle was obtained at the level of the papillary muscle in M-mode.

### 2.3 Blood pressure and heart rate measurement in mice

**FIGURE 1:**
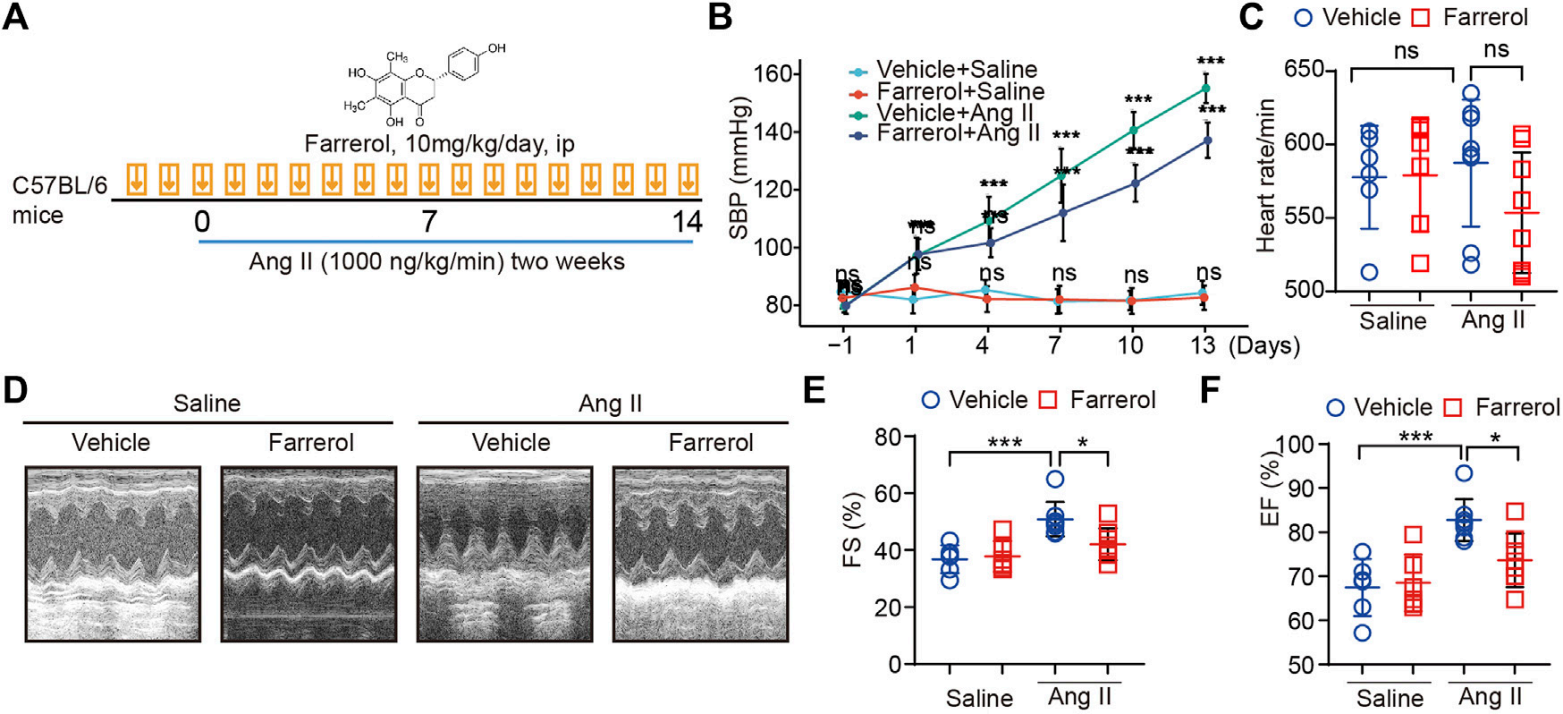
Farrerol inhibits Ang II-induced hypertension and cardiac dysfunction in mice. **(A)** Graphical flow chart; **(B)** Systolic blood pressure of mice in different treatment groups; **(C)** Heart rate of mice in different treatment groups; **(D–F)** Representative M-mode echocardiography of left ventricular (LV) chamber and echocardiographic assessment of FS(%), EF (%) (Salline = 6, Farrerol = 6, Ang II = 8, Ang II + Farrerol = 8).

Blood pressure was measured by the tailcuff method before starting treatment and every 2 days after Ang II infusion as previously described (Wang et al., 2016) (Figure 1A). Within a week before the official assay, the mice underwent training assays to acclimate them. Briefly, awake and calm mice were placed on the immobilizer with the tail fully exposed. When the sine wave is displayed on the screen of the non-invasive sphygmomanometer, press the pressure measurement button to start the measurement, record the heart rate, systolic blood pressure and diastolic blood pressure. Each mouse is measured 5 times and averaged for statistical analysis.

### 2.4 Wheat germ agglutinin (WGA) and dihydropyrimidine (DHE)

Slides were dewaxed and hydrated, washed with phosphate buffered solution (PBS) 3 times, incubated with WGA for 1 h or DHE for 30 min at room temperature, washed with PBS 3 times, seal the cover glass. Images were captured by the Nikon microscope and analysis by ImageJ.

### 2.5 Hematoxylin-eosin (H&E)

Slides were dewaxed and hydrated, washed with PBS 3 times. Placed in hematoxylin staining solution for 5 min, hydrochloric acid aqueous solution was differentiated, ammonia solution returned to blue, running water removed residual staining solution, and completed 5 min of counterstaining in eosin staining solution. Seal the cover glass. Images were captured by the Nikon microscope and analysis by ImageJ.

### 2.6 Masson’s trichrome

Slides were dewaxed and hydrated, washed with PBS 3 times, Hematoxylin staining for 5–10 min, Wash with running water for a while, and differentiate with 1% hydrochloric acid alcohol. Rinse with running water for several minutes, dye with Ponceau for 5–10 min, rinse with distilled water for a while, treat with 1% phosphomolybdic acid solution for about 5 min, counterstain with aniline blue for 5 min, seal the cover glass. Images were captured by the Nikon microscope and analysis by ImageJ.

### 2.7 Immunohistochemistry (IHC)

Slides were dewaxed and hydrated, washed with PBS 3 times, antigen retrieval, washed with PBS 3 times, 3%H_2_O_2_ 10 min, washed with PBS 3 times, blocking buffer 30 min, the primary antibody (CD68 (ARG10514) and Nitrotyrosine (ARG22269) were purchased from arigo) incubation overnight, the secondary antibody incubation at RT for 1 h. Hemotaxylin 5 min, dehydration with ethanol and xylene. Images were captured by the Nikon microscope and analysis by ImageJ.

### 2.8 Lactate dehydrogenase (LDH)

LDH were measured by Solarbio (Beijing, China) according to the manufacturer’s suggestions at 450 nm. The LDH were calculated according to the manufacturer’s suggestions.

### 2.9 Real-time PCR analysis

According to the manufacturer’s protocol, total RNA was extracted with TRIzol and RNA (500–1,000 ng) transcribed into cDNA. cDNA was used for PCR. The mRNA expression of target genes was analyzed with the 2^−ΔΔCt^ method and β-actin was used as an internal control. The sequence is showed in Table 1.

**TABLE 1:** The sequence of primers.

Gene	Forward primer (5′-3′)	Reverse primer (5′-3′)
*nppa*	GCT​TCC​AGG​CCA​TAT​TGG​AG	GGG​GGC​ATG​ACC​TCA​TCT​T
*nppb*	AGT​CCT​TCG​GTC​TCA​AGG​CA	CCG​ATC​CGG​TCT​ATC​TTG​TGC
*cola1*	TAA​GGG​TCC​CCA​ATG​GTG​AGA	GGG​TCC​CTC​GAC​TCC​TAC​AT
*cybb*	TGA​ATG​CCA​GAG​TCG​GGA​TTT	CGA​GTC​ACG​GCC​ACA​TAC​A
*actb*	GGC​TGT​ATT​CCC​CTC​CAT​CG	CCA​GTT​GGT​AAC​AAT​GCC​ATG​T

### 2.10 Western blot analysis

Radio-Immunoprecipitation Assay (RIPA) buffer was used to extract the protein in the heart tissue. The protein lysate is separated by sodium dodecyl sulfate polyacrylamide gel electrophoresis (SDS-PAGE) gel electrophoresis and transferred to a polyvinylidene fluoride (PVDF) membrane. The membrane was incubated with the antibody NOX2 (A19701) and β-actin (A3718) were purchased from ABclonal, α-SMA (ARG66381) were purchased from arigo, ERK 1/2 (11257-1-AP), p-ERK 1/2 (28733-1-AP), AKT (10176-2-AP) and p-AKT (28731-1-AP) were purchased from Proteintech), The antibodies against stat3 (ARG54150) were purchased from arigo Biolaboratories. The antibodies against p-STAT3 (9145) and NFAT2 (8032) were purchased from Cell Signaling Technology, overnight at 4°C, and then incubated with the horseradish peroxidase-conjugated secondary antibody at room temperature for 1 h. All blots are developed and labeled using electrochemiluminescence (ECL) Plus chemiluminescence system. The intensity of protein bands was measured by ImageJ. β-actin was used as an internal control.

### 2.11 Neonatal rat cardiomyocytes (NRCM) and fibroblast separation

NRCM was isolated from Sprague Dawley (SD) rats within 24 h after birth. The heart is removed and digested. The dispersed cells were then incubated on a 100 mm^3^ Petri dish in 5% CO_2_ at 37°C for 90 min. Collect unattached cardiomyocytes and transfer them to the well plate of the desired experiment. Fibroblasts continue to be cultured in 100 mm^3^. The medium required for cardiomyocytes and fibroblasts is Dulbecco’s Modified Eagle Media F12medium (DMEM/F12), 10% fetal bovine serum (FBS) and penicillin. The cells are divided into different groups according to the experiment. After Farrerol (20 μM) treatment for 24 h, Ang II (2 μM) treatment for 24 h.

### 2.12 Immunofluorescence (IF)

NRCMs or slides were permeabilized with .1% Triton X-100 for 5 min, 3% bovine serum albumin (BSA) 30 min, the primary antibody (α-actinin (A8939) was purchased from ABclonal, collagen III (22734-1-AP) was purchased from Proteintech) overnight, secondary antibody 1 h, seal the cover glass. Images were captured by the Nikon microscope and analysis by ImageJ.

### 2.13 Mitochondrial ROS measurement

Mitochondrial ROS levels were quantified by measuring the fluorescence of MitoSOX™ Red (M36008, Thermo Fisher Scientific). After treatment, cells were washed once with PBS. Then, cells were incubated with 5 μM MitoSOX™ reagent working solution at 37°C for 10 min, protected from light. Finally, the cells were rinsed three times with PBS and observed with an inverted fluorescence microscope.

### 2.14 Cell-counting-kit-8 (CCK-8)

Farrerol and Ang II was treated with fibroblast for 24 h, 48 h, 72 h, and 96 h in 96 wells. CCK-8 (K1018, APExBIO) was added and incubated for 3 h at 37°C. Optical density (OD) value was detected at 450 nm.

### 2.15 Wound-healing assay

The cells were fused to 95%–100% culture and the wound healing experiment was performed by scraping off the monolayer of cells with the tip of .1–10 μl pipette. After Farrerol and Ang II treatment, scratches were observed and photographed. ImageJ software was used for quantitative analysis of scratches.

### 2.16 Statistical analysis

Graphpad prism 9.3 software was used for statistical analysis, and all measurement data were expressed as x^2^±Standard Deviation (SD). Student’s *t*-test was used for comparison between the two groups (Vehicle vs. Farrerol), and one-way ANOVA analysis (Bonferroni’s test) was used for comparison between multiple groups (Vehicle vs. Ang II, Ang II vs. Ang II + Farrerol). Blood pressure was measured by Pairwise Comparisons of Estimated Marginal Means (www.xiantao.love/). *p < .05, **p < .01, ***p < .001, ****p < .0001 were considered statistically significant.

## 3 Results

### 3.1 Farrerol inhibits Ang II-induced hypertension and cardiac dysfunction in mice

A graphical flow chart was shown in Figure 1A. In this paper, we used Ang II to create a mouse model of cardiac hypertrophy. Ang II is known to increase blood pressure in mice. Therefore, we first detected the blood pressure of the mice in each group in this experiment. The results showed that the blood pressure of the mice was significantly increased in the Ang II group, while the blood pressure of mice was significantly decreased in Farrerol + Ang II group (Figure 1B). There was no significant difference in heart rate between groups (Figure 1C). After 14 days of Ang II treatment, cardiac function of each group was detected by ultrasound. The results showed that FS (%) and EF (%) were increased in Ang II group compared to Vehicle group, which suggested that we established a successful myocardial hypertrophy mold in mice. FS (%) and EF (%) were significantly decreased in Ang II + Farrerol group compared to Ang II group (Figures 1D–F).

### 3.2 Farrerol inhibits Ang II-induced hypertrophy in mice

**FIGURE 2:**
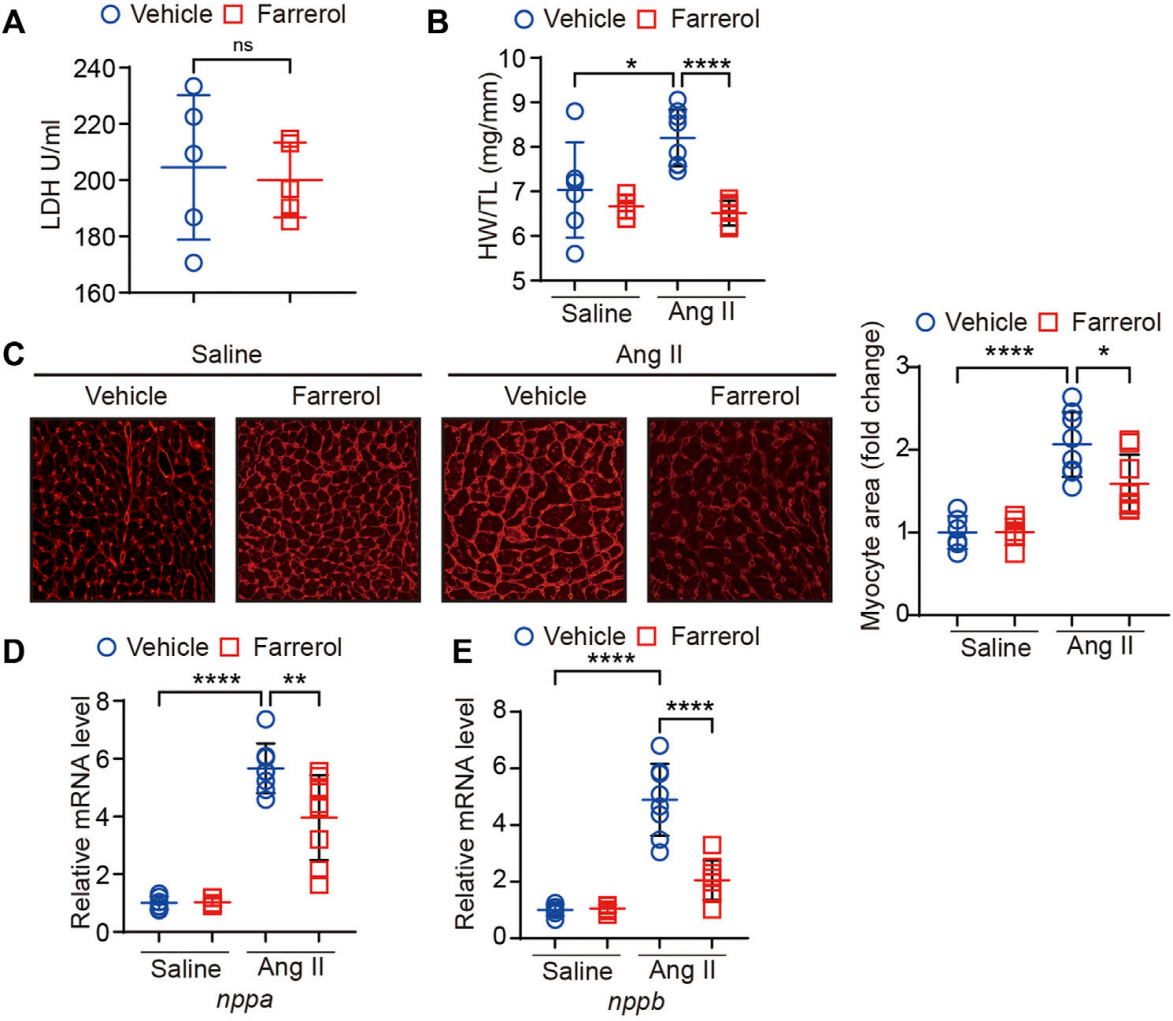
Farrerol inhibits Ang II-induced hypertrophy in mice. **(A)** The level of LDH; **(B)** Ratio of heart wright to tibia length in experimental groups; **(C)** Representative images of hearts stained with WGA and the relative value of myocardial cell size of mice in each group; **(D,E)** The mRNA level of *nppa* and *nppb* in each group (Salline = 6, Farrerol = 6, Ang II = 8, Ang II + Farrerol = 8).

We first tested whether Farrerol was toxic to the heart by means of LDH testing. The results showed no significant difference between the control group and Farrerol group (Figure 2A). We found that heart weight/tibia ratio (HW/TL) was significantly increased in the Ang II group compared to control group. The HW/TL was decreased in Farrerol + Ang II compared to Ang II group (Figure 2B). Then, WGA staining was used to observe the size level of cardiomyocytes in each group. It was found that the area of cardiomyocytes was significantly increased in the Ang II group compared with control group. The size of cardiomyocytes was decreased in Farrerol + Ang II group compared to Ang II group (Figure 2C). The mRNA expressions of cardiac hypertrophy markers *nppa* and *nppb* were further investigated in each group. It was found that the mRNA expressions of *nppa* and *nppb* were significantly increased in Ang II group compared to the control group. Farrerol could significantly reduce the mRNA expression levels of *nppa* and *nppb* induced by Ang II compared to Ang II group (Figure 2D).

### 3.3 Farrerol inhibits Ang II-induced fibrosis and inflammation in mice

**FIGURE 3:**
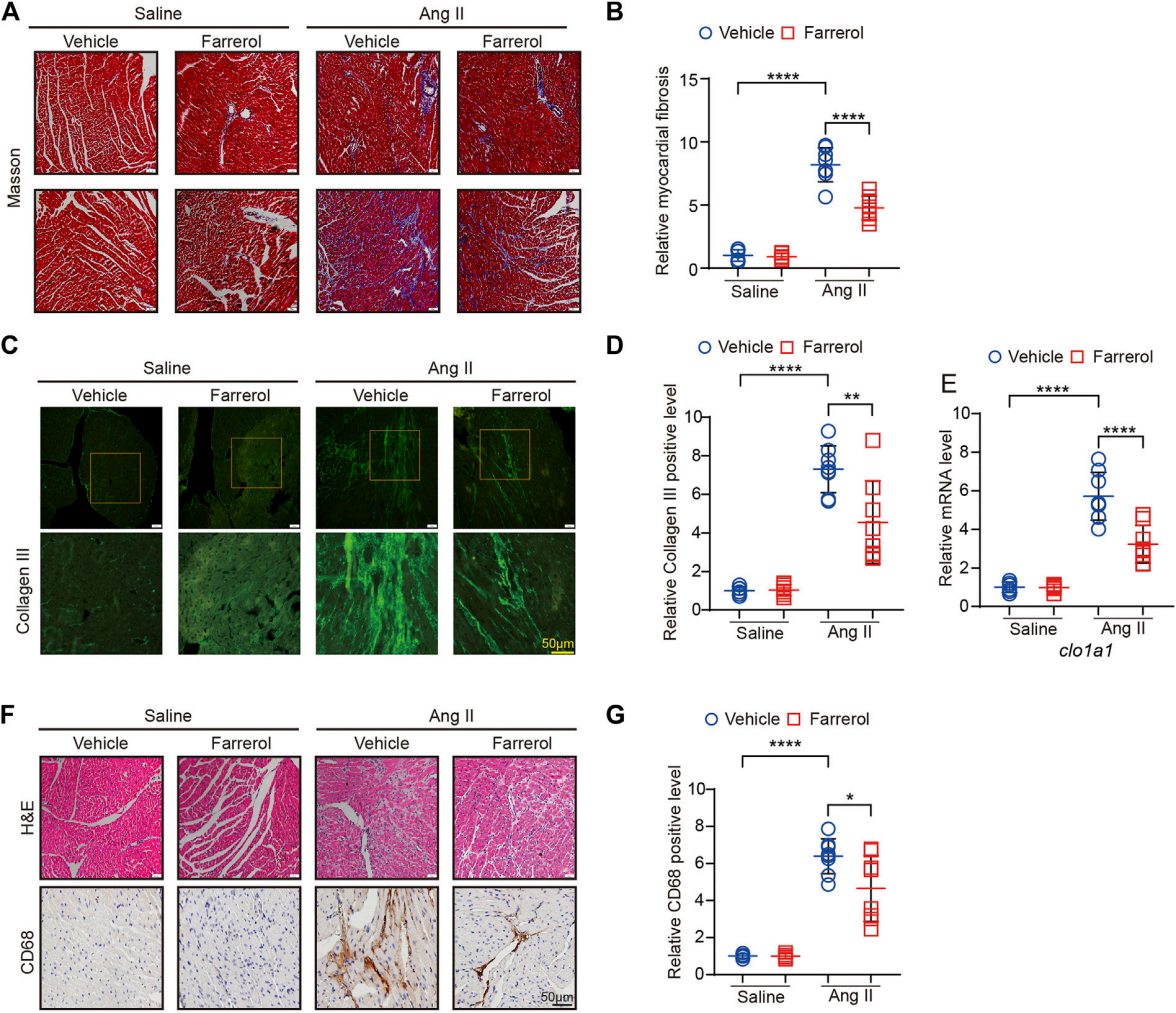
Farrerol inhibits Ang II-induced fibrosis and inflammation in mice. **(A)** Representative images of hearts stained with Masson; **(B)** The relative value of cardiac fibrosis of mice in each group; **(C)** Representative images of hearts stained with Collagen III; **(D)** The relative value of Collagen III of mice in each group; **(E)** The mRNA level of *cola1* in each group; **(F)** Representative images of hearts stained with H&E and CD68; **(G)** The relative value of CD68 of mice in each group (Salline = 6, Farrerol = 6, Ang II = 8, Ang II + Farrerol = 8).

Masson staining of myocardial tissue was used to observe the difference level of cardiac fibrosis in each group. It was found that the degree of cardiac fibrosis was significantly increased in the Ang II group compared to the control group. The level of cardiac fibrosis was decreased in Farrerol + Ang II group compared to Ang II group (Figures 3A, B). Next, we detected the fluorescence expression level of Collagen III, and the results showed that the fluorescence level of Collogen III was significantly increased in the Ang II group compared to the control group. The fluorescence level of Collagen III was significantly decreased in the Farrerol + Ang II group compared to Ang II group (Figures 3C, D). The mRNA expression of *clo1a1*, a marker of cardiac fibrosis, was further investigated in each group, and it was found that the mRNA expression of *clo1a1* was significantly increased Ang II group compared to the control group. The mRNA expression of *clo1a1* was significantly decreased in the Farrerol + Ang II group compared to Ang II group (Figure 3E). Next, H&E staining were used to observe the inflammation level in cardiac tissues of each group. As shown in Figure 3F, the degree of cardiac inflammation was significantly increased the level of nuclear aggregation in the Ang II group compared to control group. The level of nuclear aggregation was decreased in Farrerol + Ang II group compared to Ang II group. We also detected the expression of CD68 by IHC in different group. The result showed that the expression of CD68 was increased in the Ang II group compared to control group. The expression of CD68 was decreased in Farrerol + Ang II group compared to Ang II group (Figures 3F, G).

### 3.4 Farrerol inhibits Ang II-induced oxidative stress in mice

**FIGURE 4:**
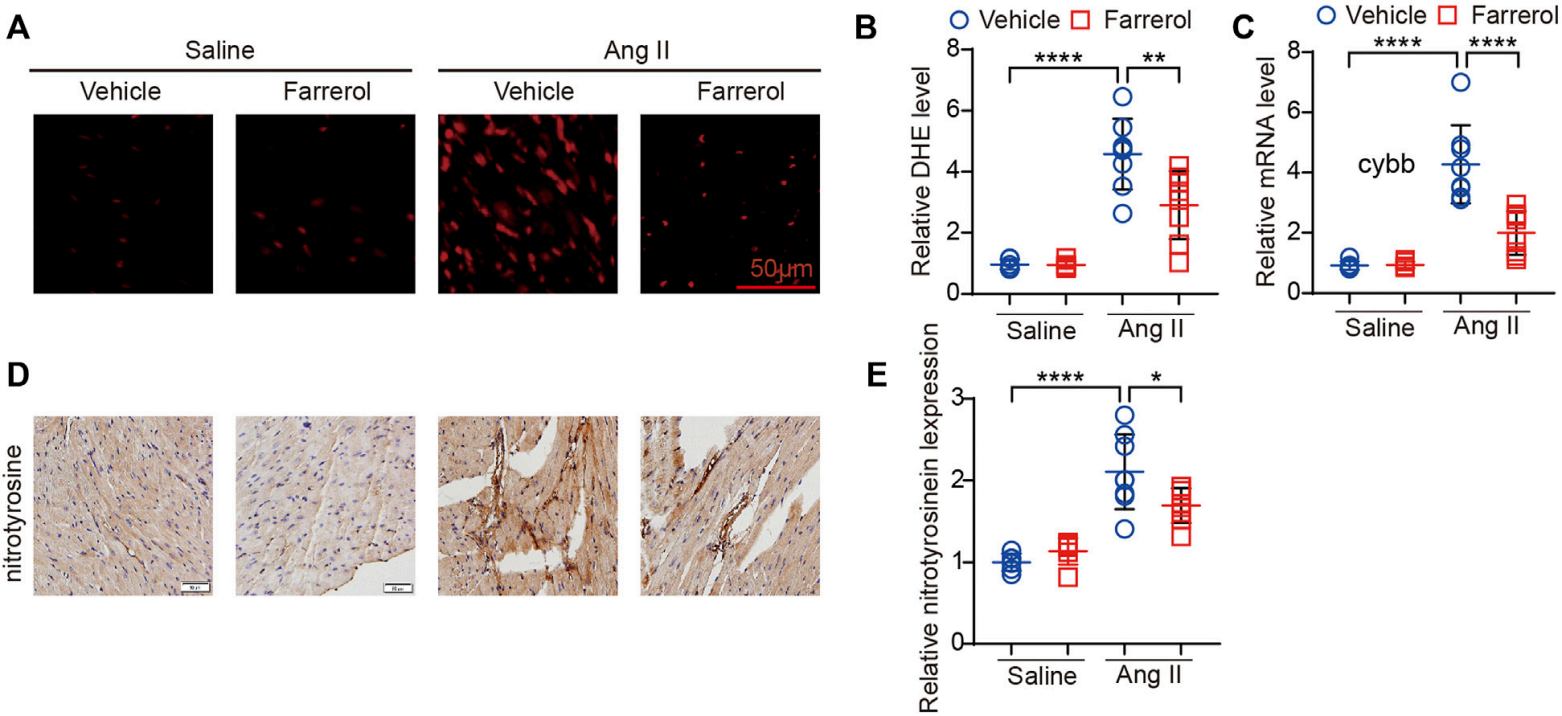
Farrerol inhibits Ang II-induced oxidative stress in mice. **(A)** Representative images of hearts stained with DHE; **(B)** The relative value of DHE of mice in each group; **(C)** The mRNA level of *cybb* in each group; **(D)** Representative images of hearts stained with nitrotyrosine; **(E)** The relative value of nitrotyrosine of mice in each group (Salline = 6, Farrerol = 6, Ang II = 8, Ang II + Farrerol = 8).

DHE staining was used to observe the oxidative stress level of heart tissues in each group. The positive rate of DHE staining fluorescence signal was significantly higher in Ang II group compared to control group, while Farrerol could reduce the positive rate of DHE staining signal in Farrerol + Ang II group compared to Ang II group (Figures 4A, B). Further we detected the mRNA expression of cardiac oxidative stress marker *cybb* in each group. The result showed that the mRNA expression of *cybb* was significantly increased in Ang II group compared to control group. Farrerol significantly reduced the mRNA level of *cybb* in Farrerol + Ang II group compared to Ang II group (Figure 4C). We also detected the expression of nitrotyrosinein by IHC. The result showed that the expression of nitrotyrosinein was increased in the Ang II group compared to control group. The expression of nitrotyrosinein was decreased in Farrerol + Ang II group compared to Ang II group (Figures 4D, E). These results suggested that Farrerol could reduce the oxidative stress level and improve the antioxidant capacity of heart tissue.

### 3.5 Effect of Farrerol on signaling pathways of cardiac hypertrophy, oxidative stress, fibrosis, and inflammation

**FIGURE 5:**
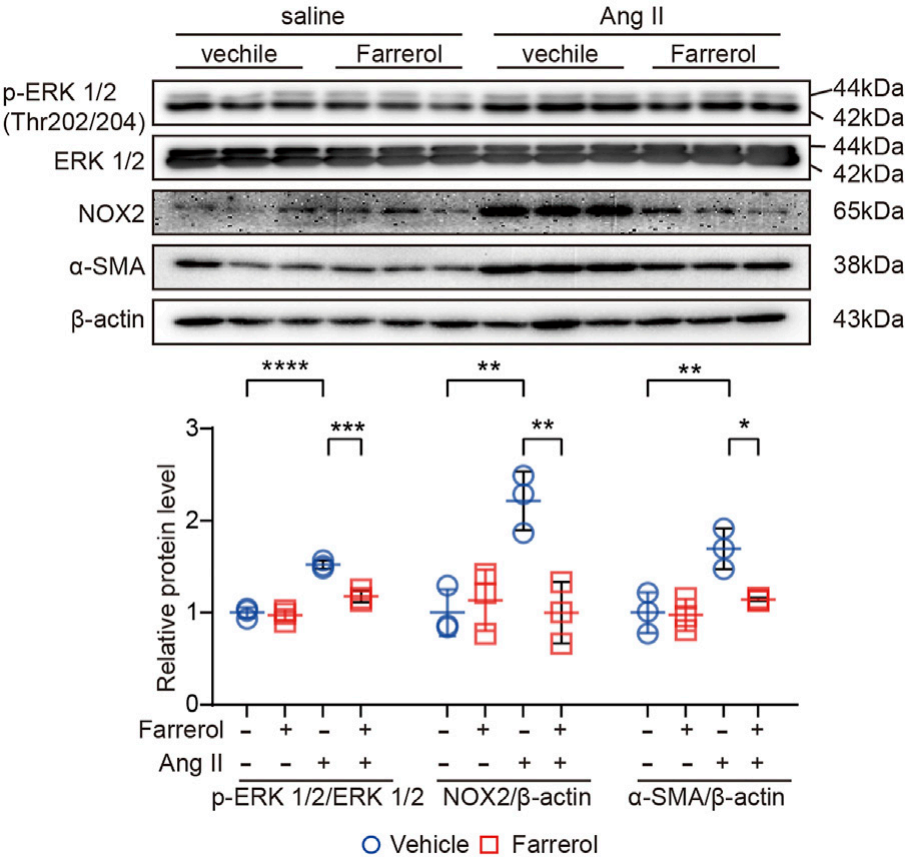
Effect of Farrerol on signaling pathways of cardiac hypertrophy, oxidative stress, fibrosis and inflammation. The protein expression of p-ERK (The202/204), ERK, NOX2, α-SMA and β-actin in the heart of each group of mice, and the relative value of the statistical chart (Salline = 3, Farrerol = 3, Ang II = 3, Ang II + Farrerol = 3).

Next, we further evaluated the expression of related-proteins in hypertrophy, inflammation and ROS signaling pathways. The results showed that the expression of p-Extracellular signal-regulated kinase [p-ERK1/2 (The202/204)], NADPH Oxidase Type 2 (NOX2) and α-smooth muscle actin (α-SMA) were significantly increased in Ang II group compared to control group. The expression levels of these proteins were significantly inhibited in Farrerol + Ang II group compared to Ang II group (Figure 5).

### 3.6 Farrerol inhibits Ang II-induced cardiac remodeling *in vitro*

**FIGURE 6:**
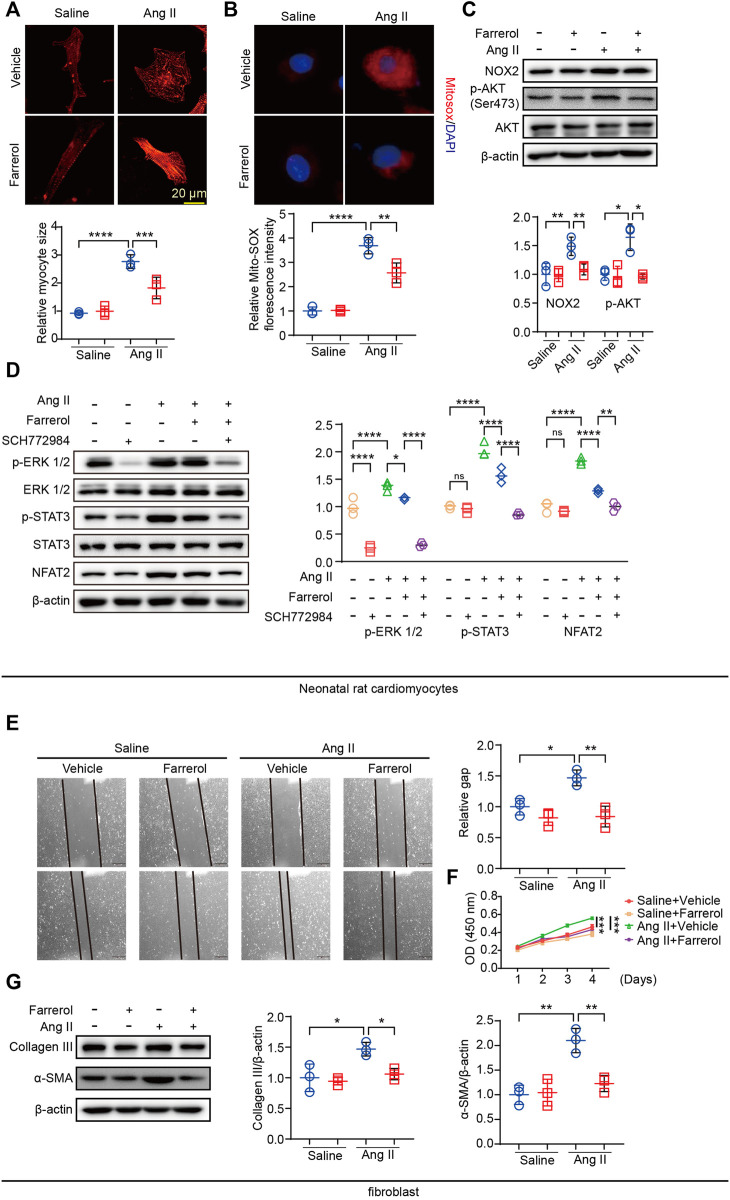
Farrerol inhibits Ang II-induced cardiac remodeling *in vitro*. **(A)** Representative images of Myocardial cells stained with α-actinin in each group and the relative value of the statistical chart; **(B)** Mitochondrial ROS levels in NRVMs were determined by MitoSOX Red staining; **(C)** The expression of p-AKT (Ser473), AKT, NOX2 and β-actin detected by Western Blot; **(D)** The expression of p-ERK 1/2, ERK 1/2, p-STAT3, STAT3, NFAT2 and β-actin detected by Western Blot; **(E)** the migration ability of fibroblasts by Wound scratch assay in each group; **(F)** The proliferation ability of fibroblasts was detected by CCK-8; **(G)** The expression of p-AKT (Ser473), AKT, NOX2 and β-actin detected by Western Blot (Salline = 3, Farrerol = 3, Ang II = 3, Ang II + Farrerol = 3).

We have established that Farrerol regulated cardiac remodeling *in vivo*, and then we studied the effect of Farrerol (20 μM) on NRCMs and fibroblast *in vitro*. The cell area was significantly larger in Ang II group compared to control group in NRCM. Farrerol reduced cell surface area in Farrerol + Ang II group compared to Ang II group in NRCM (Figure 6A). In addition, we used a MitoSOX Red probe to measure mitochondrial ROS levels in NRVMs. The results showed that Farrerol attenuated the increase in Ang II-induced mitochondrial ROS levels (Figure 6B). Next we examined the expression of hypertrophy and oxidative stress related proteins. The results showed that, the expression levels of p-AKT (Ser473) and NOX2 were significantly increased in Ang II group compared to the control group in NRCM. The expression levels of p-AKT (Ser473) and NOX2 were significantly decreased in Farrerol + Ang II group compared to Ang II group in NRCM (Figure 6C). We also examined the expression of downstream cardiac hypertrophy signaling proteins through MAPK-ERK pathway. The results showed that the expressions of p-ERK 1/2 were significantly increased after treatment with an inhibitor of ERK (SCH772984, S7101, SELLECK), but not p-STST3 and NFAT2. The expressions of p-ERK 1/2, p-STST3 and NFAT2 were significantly increased after the addition of Ang II stimulation, while the expressions of these proteins were significantly decreased after Farrerol treatment compared with Ang II group. After treatment with SCH772984, the expression of these proteins was further decreased in NRCM (Figure 6D).

Cardiac remodeling includes proliferation and migration of fibroblasts in addition to myocardial hypertrophy. We next examined the effect of Farrerol on fibroblasts. The migration ability of fibroblasts was detected by Wound-healing assay. The results showed that it was significantly promoted the migration of fibroblasts in Ang II group compared with the control group, while Farrerol significantly inhibited the migration of fibroblasts in Farrerol group compared to control group (Figure 6E). We detected the effect of Farrerol on fibroblast proliferation by CCK-8. The results showed that it was significantly promoted the proliferation of fibroblasts in Ang II group compared to the control group, while Farrerol significantly inhibited the proliferation of fibroblasts in Farrerol group compared to the control group (Figure 6F). Next, Western Blot was used to detect the expression of Collagen III and α-SMA in each group. The results showed that Ang II significantly promoted the expression of Collagen III and α-SMA compared to control group, while the expression of Collagen III and α-SMA were significantly inhibited in Ang II + Farrerol compared to Ang II group (Figure 6G).

## 4 Discussion

The process of ardiac remodeling is carried out when the heart is damaged or has an abnormal hemodynamic condition. This reconstruction process leads to changes in the heart’s size, shape, and function. (Verdejo et al., 2012). During the process of myocardial reconstruction, interstitial fibrosis develops can reduce the heart’s cardiac compliance. It also affects the heart’s contractile function. This condition can lead to a reduction in the number of myocardial cells and to apoptosis (Pauschinger et al., 2004; Turner, 2018). In addition, collagen fibers can also wrap around the cardiomyocytes, which can lead to electrical conduction disorders and cause arrhythmia. This condition can also decrease the blood vessel density (Widiapradja et al., 2017; Zhang and Shah, 2007). Therefore, it is necessary to deeply study the molecular regulatory mechanism of cardiac remodeling in order to discover new targets and to improve heart failure treatment.

Despite a large number of studies on cardiac remodeling, the specific mechanism that underlies the phenomenon is still unclear. Apoptosis of cardiomyocytes and proliferation of non-cardiomyocytes (fibroblasts, endothelial cells, smooth muscle cells, etc.), as well as myocardial inflammation, oxidative stress, fibrosis, and intramyocardial angiogenesis have all been shown to be associated with myocardial remodeling.

Clinical and experimental studies have shown that Ang II is a major effector of the RAAS and plays an important role in the biological process leading to cardiac remodeling. In addition to its physiological role in the regulation of arterial blood pressure (that is, through vasoconstriction and sodium-water retention), Ang II also directly induces cardiac remodeling by activating oxidative stress, inflammation, hypertrophy, fibrosis, and extracellular matrix accumulation (Liu et al., 2017; Zhang et al., 2017).

In the past few decades, there has been evidence that certain Chinese herbal medicines are effective in treating human cardiovascular diseases, which can promote the activation of cellular inflammation, oxidative stress and other related signaling pathways involved in human cardiovascular disease at the molecular level (Chang et al., 2020; Zhao et al., 2020).

Farrerol is a kind of plant polyphenol compound that is abundant in some fruits, vegetables and herbal plants. According to past studies, it has many therapeutic effects on various pathological conditions, such as cancer, muscle atrophy, inflammation, microbial infections, and oxidative stress (Guo et al., 2022; Wu et al., 2022). These therapeutic effects of Farrerol are mainly due to free radical scavenging, antioxidant and anti-inflammatory properties. Farrerol alleviates myocardial ischemia/Reperfusion injury *in vivo* (Zhou et al., 2022). We believe that Farrerol plays the same role in cardio-protection from hypertrophy and remodeling.

In this study, we found that Farrerol can inhibit the expression of *nppa* and *nppb*, which are markers of cardiac hypertrophy. Therefore, it can be concluded that Farrerol may effectively protect against cardiac hypertrophy caused by Ang II. Cardiomyocyte hypertrophy is an important indicator of cardiac remodeling in mice. We have demonstrated that Farrerol can inhibit the increase in size of cardiomyocytes induced by Ang II *in vivo* and *in vitro*.

Inflammation and fibrosis are common pathological attributes of cardiac remodeling, and are related to structural damage and impaired function in the left ventricle (Fu et al., 2022). Studies have shown that the increased expression of proinflammatory factors promotes cardiac remodeling. The initiation of the inflammatory response causes the accumulation of extracellular matrix and accelerates the process of myocardial fibrosis (Cooper et al., 2022). Oxidative stress is a key element that affects cardiac remodeling (Mahmoudi et al., 2008). The relationship between Farrerol and oxidative stress has been studied. Farrerol can affect the occurrence and development of diseases by regulating NOX4/ROS/ERK/TGF-β signaling pathway, NRF2/Keap1 signaling pathway, HO-1 and NQO1 signaling pathway (Qin et al., 2022; Chen et al., 2020; Ma et al., 2019). In this paper, we found that Farrerol could inhibit the expression of DHE, mitoSOX and the expression of NOX2. Thus, we found that Farrerol protected heart tissue from inflammation, fibrosis and oxidative stress.

Studies have shown that activation of MAPK signaling induces cardiac hypertrophy. Specific MAPK/ERK inhibitors attenuate this process. STAT3 and NFAT2 play key roles in the development of cardiac hypertrophy. As shown in the results above, Ang II can promote the expression of p-ERK, p-STAT3, and NFAT2, while Farrerol and ERK inhibitors can significantly inhibit their expression, and we found that Farrerol may exert an anti-cardiac hypertrophy effect through MAPK/ERK. In subsequent experiments, we could overexpress the expression levels of MAPK/ERK pathway-related proteins, which further proved that Farrerol may exert its anti-cardiac hypertrophy effect through MAPK/ERK.

**FIGURE 7:**
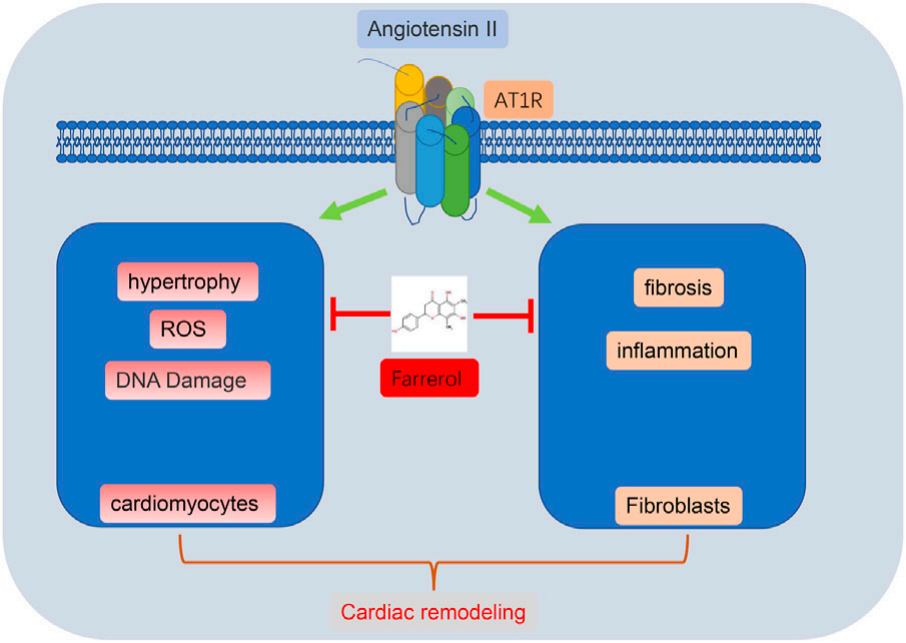
Flow chart of Farrerol inhibition of Ang II-induced cardiac remodeling.

In summary, Farrerol could effectively inhibit Ang II-induced myocardial remodeling and myocardial hypertrophy in mice, indicating that Farrerol may be a candidate drug for the treatment of myocardial remodeling (Figure 7).

## Data Availability

The raw data supporting the conclusion of this article will be made available by the authors, without undue reservation.
